# The natural drug DIAVIT is protective in a type II mouse model of diabetic nephropathy

**DOI:** 10.1371/journal.pone.0212910

**Published:** 2019-03-13

**Authors:** Megan Stevens, Christopher R. Neal, Elena C. Craciun, Maria Dronca, Steven J. Harper, Sebastian Oltean

**Affiliations:** 1 Institute of Biomedical and Clinical Sciences, University of Exeter Medical School, Exeter, United Kingdom; 2 School of Physiology and Pharmacology, University of Bristol, Bristol, United Kingdom; 3 Bristol Renal, School of Clinical Sciences, University of Bristol, Bristol, United Kingdom; 4 Department of Pharmaceutical Biochemistry and Clinical Laboratory, School of Pharmacy, University of Medicine and Pharmacy “Iuliu Hatieganu” Cluj-Napoca, Romania; 5 Department of Medical Biochemistry, School of Medicine, University of Medicine and Pharmacy “Iuliu Hatieganu” Cluj-Napoca, Romania; Institute of Subtropical Agriculture, Chinese Academy of Sciences, CHINA

## Abstract

There is evidence to suggest that abnormal angiogenesis, inflammation, and fibrosis drive diabetic nephropathy (DN). However, there is no specific treatment to counteract these processes. We aimed to determine whether DIAVIT, a natural *Vaccinium myrtillus* (blueberry) and *Hippophae Rhamnoides* (sea buckthorn) extract, is protective in a model of type II DN. Diabetic db/db mice were administered DIAVIT in their drinking water for 14 weeks. We assessed the functional, structural, and ultra-structural phenotype of three experimental groups (lean+vehicle, db/db+vehicle, db/db+DIAVIT). We also investigated the angiogenic and fibrotic pathways involved in the mechanism of action of DIAVIT. Diabetic db/db mice developed hyperglycaemia, albuminuria, and an increased glomerular water permeability; the latter two were prevented by DIAVIT. db/db mice developed fibrotic glomeruli, endothelial insult, and glomerular ultra-structural changes, which were not present in DIAVIT-treated mice. Vascular endothelial growth factor A (VEGF-A) splicing was altered in the db/db kidney cortex, increasing the pro-angiogenic VEGF-A_165_ relative to the anti-angiogenic VEGF-A_165_b. This was partially prevented with DIAVIT treatment. Delphinidin, an anthocyanin abundant in DIAVIT, increased the VEGF-A_165_b expression relative to total VEGF-A_165_ in cultured podocytes through phosphorylation of the splice factor SRSF6. DIAVIT, in particular delphinidin, alters VEGF-A splicing in type II DN, rescuing the DN phenotype. This study highlights the therapeutic potential of natural drugs in DN through the manipulation of gene splicing and expression.

## Introduction

Diabetic nephropathy (DN) is the leading cause of end stage renal disease (ESRD) in the USA and across the world, affecting 50% of diabetic patients [1–3]. Glycaemic control, lipid and blood pressure control, plus renin-angiotensin-aldosterone system (RAAS) blockade are the current treatments of choice [4], but many DN patients still progress to ESRD. Therefore, novel therapeutic approaches for the treatment of DN are required.

In DN, alterations of the glomerular filtration barrier (GFB) result in increased permeability to protein; such changes include glomerular basement membrane (GBM) thickening, mesangial matrix expansion (MME), podocyte detachment, and glomerular endothelial cell damage [5,6]. An increasing number of studies suggest that angiogenesis, inflammation, and fibrosis are responsible for the onset of type II DN [7,8]. Abnormal expression of vascular endothelial growth factor A (VEGF-A) in the kidney has been widely reported in DN [9–10]. Alternative splicing of exon 8 of VEGF-A results in an anti-angiogenic splice isoform, VEGF-A_165_b [11], which is protective in DN and renal disease [7,12]. In addition, activation of the transcription factor p65 nuclear factor kappa B (p65-NFĸB) is linked to the regulatory pathways that underlie the pro-inflammatory and pro-fibrotic response [13], and an increase in p65-NFĸB translocation to the nucleus has been shown in human DN [14].

In diabetes, glucotoxicity results in the generation of free radicals and oxidative stress, leading to the progression of diabetic complications [15]. Activation of NFĸB is widely reported to be evoked by increased oxidative stress [16]. Previous studies in rodent models of DN have indicated that a reduction in oxidative stress using anti-oxidants, such as those found in red berry extracts, resulted in decreased NFĸB activity, thus improving kidney function [17,18]. Other studies have also found that berry/polyphenol rich extracts protect against fibrosis, angiogenesis, and inflammation in the kidneys of diabetic animal models [19–21].

DIAVIT is a natural drug based on polyphenol-rich blueberry (*Vaccinium myrtillus*) and sea buckthorn (*Hippophae Rhamnoides*), which has been approved as an adjunct therapy for diabetes in Romania. DIAVIT contains approximately 10 mg anthocyanins per gram, which have been previously described to decrease vessel permeability, improve microcirculation and exert antioxidant activity [22,23]. The most abundant is delphinidin, which has been previously reported to have potent anti-angiogenic properties through the inhibition of PI3K/Akt/mTOR signaling pathways [24,25].

The aim of the present study was to test the hypothesis that DIAVIT would have a beneficial effect on the phenotype of diabetic renal lesions in the db/db mouse model of type II DN. Furthermore, we aimed to determine by which mechanism DIAVIT was exerting a protective effect by investigating pathways linked to the pro-angiogenic and pro-fibrotic response, as well as assessing the mechanistic effect of the most abundant anthocyanin present in DIAVIT, delphinidin, on the expression and splicing of VEGF-A.

## Materials and methods

### Ethics approval

All experiments and procedures were approved by the UK Home office in accordance with the Animals (Scientific Procedures) Act 1986, and the Guide for the Care and Use of Laboratory Animals was followed.

### Administration of DIAVIT

DIAVIT tablets (1 g) were obtained from Plantarom Laboratories, Cluj, Romania (Lot: 01, Expiry: January 2017). One tablet was ground to a fine powder and dissolved into 250 ml of drinking water. This dose was equivalent to the highest recommended daily dose of DIAVIT per kg body weight in patients, which equated to approximately 6 g/kg/day. The water was changed three times weekly. Lean and non-diabetic db/db mice consumed approximately 5 ml water per day, whereas diabetic mice consumed up to 60 ml per day, depending of the severity of diabetes. Information regarding the extract dosage of DIAVIT can be found in Table 1. We performed gas chromatography and mass spectrometry (GC-MS) to determine the complex chemical composition of the extract (S1 Fig). A large part of the extract in the DIAVIT tablets are dried blueberries from Romania—the composition of the extracts from these blueberries, especially anthocyanins content, has been extensively analysed before [26, 27].

**Table 1:** Dietary dosage of DIAVIT.

DIAVIT composition	Per tablet (mg)	Daily dosage (g/kg)
*Vaccinium myirtillus*	392	2.35
*Hippophae rhamnoides*	167	1.00

### Animal functional studies

Male *BKS*.*Cg-+Lepr*^*db*^*/+Lepr*^*db*^*/OlaHsd* (db/db; obtained from Envigo) and lean control mice were obtained from Envigo (UK) (5 weeks; 25–49 g). Blood glucose was measured via blood collection from the tail vein, which was applied to an ACCU-CHEK strip (ACCU-CHEK, Roche) to determine the concentration in mmol/l. Mice were deemed diabetic if they had two consecutive blood glucose readings >15 mmol/l taken 48 h apart. Baseline urine, weight, and blood glucose measurements were taken at 6 weeks of age, and DIAVIT administration into the drinking water began immediately after. Urine collection, blood glucose measurement, and animals weights were done every week up until 20 weeks of age (week 14 of experiment), when they were killed. There were three groups of mice; lean (n = 6), db/db (n = 9), and db/db treated with DIAVIT (n = 9). Statistical power calculations showed that six control and eight experimental mice were needed to see a statistical difference in the functional phenotype (p>0.05) with a power value of 0.80 (>80%).

The urinary albumin creatinine ratio (uACR) was used as a measure of protein loss in the urine. Albumin was quantified with an albumin ELISA (Bethyl Laboratories, Inc), and creatinine with an enzymatic spectrophotometric assay (Creatinine Companion, Exocell). Assays were repeated in triplicate for each time point. Upon culling of mice via cervical dislocation, blood was collected for plasma creatinine measurements (Creatinine Companion, Exocell). Kidneys were removed and part of the cortex was immediately diced into 1 mm^3^ pieces and fixed in 2.5% gluteraldehyde in 0.1 M cacodylate buffer for electron microscopy (EM). Pieces of kidney cortex were also fixed in 4% paraformaldehyde (PFA), snap frozen in embedding medium (OCT), and snap frozen in liquid nitrogen before storing at -80°C for RNA and protein analysis at a later date. The remaining kidney was used to harvest glomeruli, using a standard sieving technique, for use an oncometric assay to determine the glomerular water permeability normalised to glomerular area (L_p_A/V_i_) of individual glomeruli *ex vivo* [28]. L_p_A/V_i_ experiments were carried out on 6 mice per group, 3–5 glomeruli per mouse.

### Structural and ultra-structural phenotype

PFA-fixed kidney cortex was sectioned at 5 μm thickness and stained with Periodic Acid Schiff (PAS) and Masson’s Trichrome Blue stain (both Sigma). Sections were imaged on a light microscope and experiments were repeated three times on 4 mice per group.

The ultra-structural phenotype was determined by post-fixation of the glutaraldehyde-fixed diced kidney cortex with 1% osmium tetroxide, before embedding in Araldite (Agar Scientific). Sections were cut at 50-100nm thickness and stained with 3% aqueous uranyl acetate and Reynolds’ lead citrate solution. After images were taken, detailed measurements of the filtration barrier were taken by a blinded experimenter at random points using ImageJ. Measurements included GBM width, number of endothelial fenestrations and podocyte foot processes per μm length, and average podocyte foot process width. We also assessed whether there was evidence of glomerular MME.

### Cell culture studies

*Mycoplasma*-free immortalized human podocytes (hTERT; Evercyte) were plated in to 12-well plates before treating for 48 hrs with DIAVIT (1 mg/ml in 1xPBS) or delphinidin chloride (Sigma Aldrich) (10 μg/ml in DMSO). A dosage of 1 mg/ml DIAVIT was chosen because this equated to an anthocyanin concentration of approximately 10 μg/ml, of which delphinidin is the most abundant in the extract, which has been shown to have anti-angiogenic properties in previous studies [25].

To determine the effect of delphinidin on the phosphorylation of SR proteins, podocytes were serum starved for 2 hrs before treating with delphinidin chloride (10 μg/ml), or DMSO control for a further 2 hrs. Cells were then stimulated with a diabetic soup (30 mM Glucose, 1 ng/ml TNFα, 1 ng/ml IL-6, and 100 nM insulin) or an osmotic control (5mM Glucose + 25 mM Mannitol) for 0.5 or 1 hrs, before extracting the protein.

### Matrigel angiogenesis assay

Podocytes were treated with either DIAVIT (1 mg/ml) or DIAVIT plus anti-VEGF-A_165_b (1 μg/ml), or delphinidin chloride (10 μg/ml) or delphinidin plus anti-VEGF-A_165_b (1 μg/ml) for 48 hrs. IgG was added to controls. The conditioned media was then used to treat human umblical vein endothelial cells (HUVECs) plated onto Matrigel. The wells were imaged 4 hrs later and the tubule length and number of branch points were quantified.

### Immunofluorescence

Frozen kidney cortex was sectioned at 5 μm thickness, mounted on to glass slides, and fixed for 10 min with 4% PFA before washing in PBS. Sections were blocked with 3% bovine serum albumin (BSA) and 5% normal goat serum in PBS for 1 hr before incubating with the primary antibody (anti-collagen IV or anti-fibronectin, 1:100, Abcam; anti-nephrin, 1:250, Acris; anti-podocin, 1:250, Sigma; anti-PECAM-1, 1:100, BD Bioscience) diluted in 3% BSA in PBS at 4°C overnight. After washing in PBS, the appropriate fluorescent secondary antibody was used (Alexa Fluor) in 3% BSA in PBS for 2 hr at room temperature. Sections were then washed in PBS before staining with DAPI and mounting with coverslips. Glomeruli and cortex were imaged using a fluorescent microscope. Experiments were repeated three times on 3–4 mice per group.

### Western blotting

Denatured protein samples were run on mini-PROTEAN TGX Stain Free pre-cast gels (4–15%, BIORAD), which allow for visualisation and accurate analysis of the total protein loaded for each sample using a Gel-Doc EZ (BIO-RAD) imaging system. The use of this system means a housekeeping protein loading control is not required as the amount of protein on the membrane for each sample can be quantified. Once protein had been transferred on to a PVDF membrane, total protein could be quantified. Membranes were blocked in 3% BSA in TBS plus 0.3% Tween before being probed with either anti-collagen IV (Abcam), anti-fibronectin (Abcam), anti-nephrin, Acris), anti-podocin (Sigma), anti-VEGF receptor 2 (VEGFR2) (Cell Signalling), anti-VEGF A20 (Santa Cruz), anti-mVEGF-A_165_b (Prof Kenneth Walsh, Boston University), anti-phospho-Akt^Ser473^ (Cell Signalling), anti-Akt (Cell Signalling), anti-phospho-extracellular signal-related kinase 1/2 (ERK1/2) (Cell Signalling), anti-ERK1/2 (Cell Signalling), anti- cyclooxygenase 2 (COX-2) (Cell Signalling), anti-wilms tumor 1 (WT1) (Abcam), anti-p65-nuclear factor kappa B (NFĸB) (Cell Signalling), anti-SR proteins 1H4 (Santa Cruz), and anti-phospho-SR proteins (mab104), all at 1:1000 dilution in 3% BSA-TBS-Tween (0.3%), at 4°C overnight. After washing membranes in TBS-Tween (0.3%), HRP-conjugated secondary antibodies were diluted in 3% BSA-TBS-Tween (0.3%), 1:10,000. Membranes were washed again and imaged using ECL detection agent (BIO-RAD) on an Amersham imager. The protein of interest was then normalised to the total protein loaded for each sample, as quantified by the Gel-Doc EZ imaging system (BIO-RAD). Experiments were repeated on at least three biological repeats, with the relative controls run on the same blot.

### Statistical analysis

We performed statistical analysis using GraphPad Prism software. Data was tested for normality and either a one-way or two-way ANOVA was used to analyse data sets. Post-hoc analysis was then carried out using the Bonferroni test for comparison between pairs. All results are presented as the average ± standard error of the mean (SEM). Imaging and analysis was blinded to the researcher to restrict bias. Details of biological and technical repeats can be found in the methods section. P values <0.05 were considered statistically significant. Throughout the manuscript, * indicates a significant different between lean mice and db/db mice and † indicates a significant difference between db/db mice and db/db + DIAVIT mice, as determined by a one-way ANOVA with Bonferroni post-hoc test.

## Results

### DIAVIT is protective against diabetes-induced increases in glomerular water permeability and albuminuria

**Fig 1:**
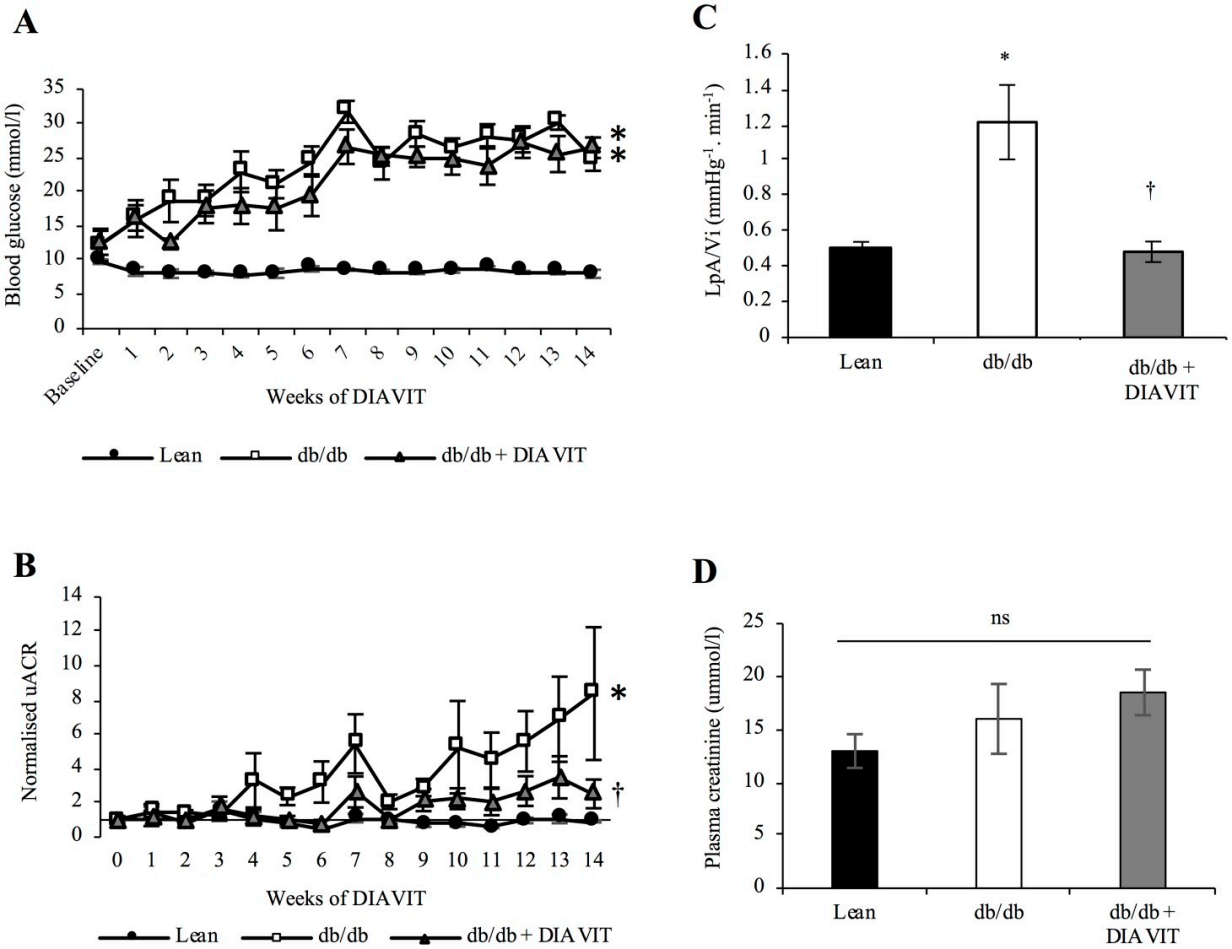
DIAVIT prevents albuminuria and increased glomerular water permeability in diabetic db/db mice, whilst having no effect on blood glucose. **A)** db/db mice develop increased blood glucose at 6–7 weeks of age (week 1 of treatment), compared to lean controls (*p<0.05), which is not affected by DIAVIT consumption (ns; p>0.05; n = 6–9 mice; Two-way ANOVA based on the average values over 14 weeks). **B)** When assessing the urinary albumin creatinine ratio (uACR), db/db mice develop progressive albuminuria at 10 weeks of age (week 4 of treatment), compared to lean controls (*p<0.05), which is significantly rescued by DIAVIT (†p<0.05; n = 6–9 mice; Two-way ANOVA based on the average values over 14 weeks). **C)** Glomeruli from db/db mice have an increased glomerular water permeability (L_p_A/V_i_) when compared to lean control glomeruli (*p<0.05), which is significantly rescued in glomeruli from DIAVIT-treated db/db mice (†p<0.05; n = 6 mice, 15–25 glomeruli; One-way ANOVA with Bonferroni post-hoc test for comparison between pairs). **D)** Plasma creatinine levels remained unchanged between groups (ns; p>0.05; n = 6–9 mice; One-way ANOVA with Bonferroni post-hoc test for comparison between pairs).

Both db/db and db/db + DIAVIT mice developed and maintained a significant increase in their blood glucose levels one week after beginning of the study, compared to lean controls (Fig 1A; blood glucose averaged over 14 weeks: 8.29 ± 0.08, 24.54 ± 1.23, and 21.83 ± 1.28 mmol/l in lean, db/db, and db/db + DIAVIT mice, respectively). There was no significant difference in blood glucose levels between the db/db and db/db + DIAVIT groups. However, DIAVIT treatment did prevent the progressive increase in albuminuria observed in db/db mice (Fig 1B; uACR (μg/mg) at 14 weeks: lean, 12.8 ± 1.2 μg/mg; db/db, 312.3 ± 154.4 μg/mg; db/db + DIAVIT, 138.7 ± 19.4 μg/mg; values normalized to baseline shown in S2 Fig). Furthermore, the water permeability of individual glomeruli *ex vivo* was significantly increased in db/db mice after 14 weeks of elevated blood glucose compared to lean controls (Fig 1C; *p<0.05). This was significantly prevented in db/db + DIAVIT glomeruli (†p<0.05). Although db/db mice developed type II diabetes and albuminuria, no changes in the plasma creatinine levels were observed (Fig 1D). Therefore, DIAVIT protected against increases in glomerular water permeability and albuminuria in type II diabetic mice without altering glycaemia.

### DIAVIT prevents the development of diabetes-induced renal fibrosis

**Fig 2:**
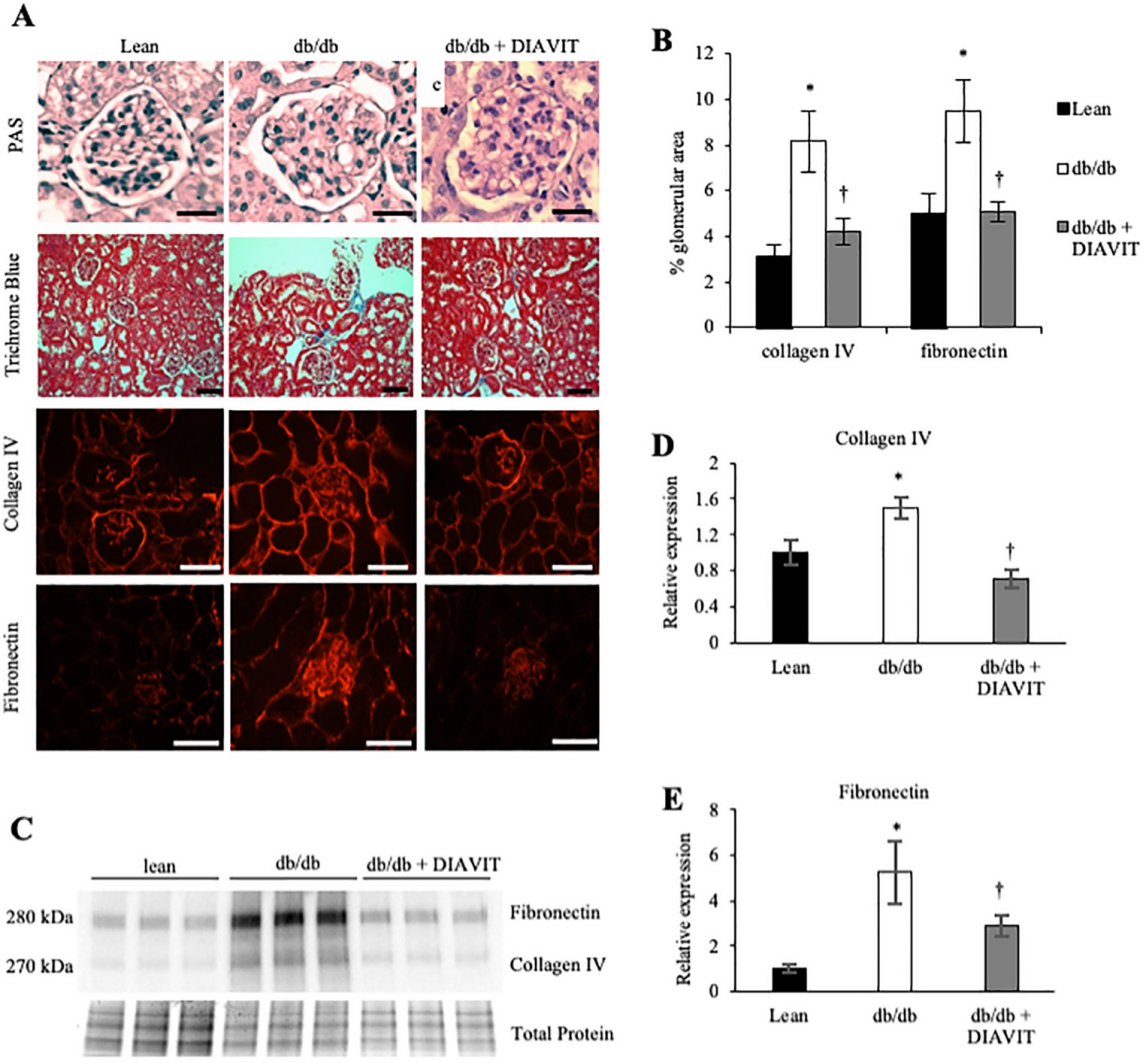
Diabetic db/db mice develop glomerular fibrosis, which is prevented in DIAVIT treated db/db mice. **A)** Periodic Acid Schiff (PAS) staining indicated structural abnormalities in db/db glomeruli, including mesangial matrix expansion and the presence of vacuoles (scale bar 40 μm). These appeared to be less frequent in DIAVIT-treated db/db glomeruli. Trichrome blue staining showed an increase in fibrosis (blue collagen staining) in the db/db kidney cortex, which was lower in the DIAVIT-treated db/db kidneys (scale bar 40 μm). More specifically, immunofluorescence for collagen IV and fibronectin showed an increase in glomerular fibrosis in db/db mice, compared to lean controls, which was prevented by DIAVIT treatment of the diabetic mice (quantified in **B**; *p<0.05 lean vs db/db; †p<0.05 db/db vs db/db + DIAVIT; n = 4 mice; 15–20 glomeruli per mouse; One-way ANOVA with Bonferroni post-hoc test for comparison between pairs; scale bar is 40 μm). **C)** Western blotting of protein from the kidney cortex confirms an increase in the protein expression of collagen IV and fibronectin in db/db mice, which is prevented with DIAVIT treatment. Analysis of the Western blots is summarised in **D** and **E** (*p<0.05 lean vs db/db; †p<0.05 db/db vs db/db + DIAVIT; n = 3–4 mice; One-way ANOVA with Bonferroni post-hoc test for comparison between pairs).

After 14 weeks of DIAVIT treatment the structural phenotype was assessed. PAS staining indicated some MME and the development of vacuoles within the glomeruli of db/db mice (Fig 2A), which appeared to be reduced when db/db mice had been treated with DIAVIT. In addition, Trichrome blue staining showed an increase in collagen (blue) deposition in the glomeruli and cortex of db/db kidneys (Fig 2A), which also appeared to be reduced in db/db + DIAVIT mice. To quantify the extent of glomerular fibrosis, we carried out immunofluorescence for collagen IV and fibronectin; two proteins highly expressed in a fibrotic kidney (Fig 2A). Both collagen IV and fibronectin expression were increased in the glomeruli of diabetic db/db mice compared to lean controls (Fig 2B; *p<0.05). However, they were significantly reduced in DIAVIT-treated db/db glomeruli (†p<0.05).

The increase in kidney fibrosis observed in db/db mice was further assessed by Western blotting of kidney cortex proteins for collagen IV and fibronectin. The relative expression of both proteins was increased in db/db kidneys compared to lean controls (*p<0.05), which was significantly prevented in db/db + DIAVIT kidneys (†p<0.05) (Fig 2C, 2D and 2E). Therefore, DIAVIT protects against renal fibrosis in a type II model of diabetes.

### DIAVIT protects against the endothelial damage caused by type II diabetes

**Fig 3:**
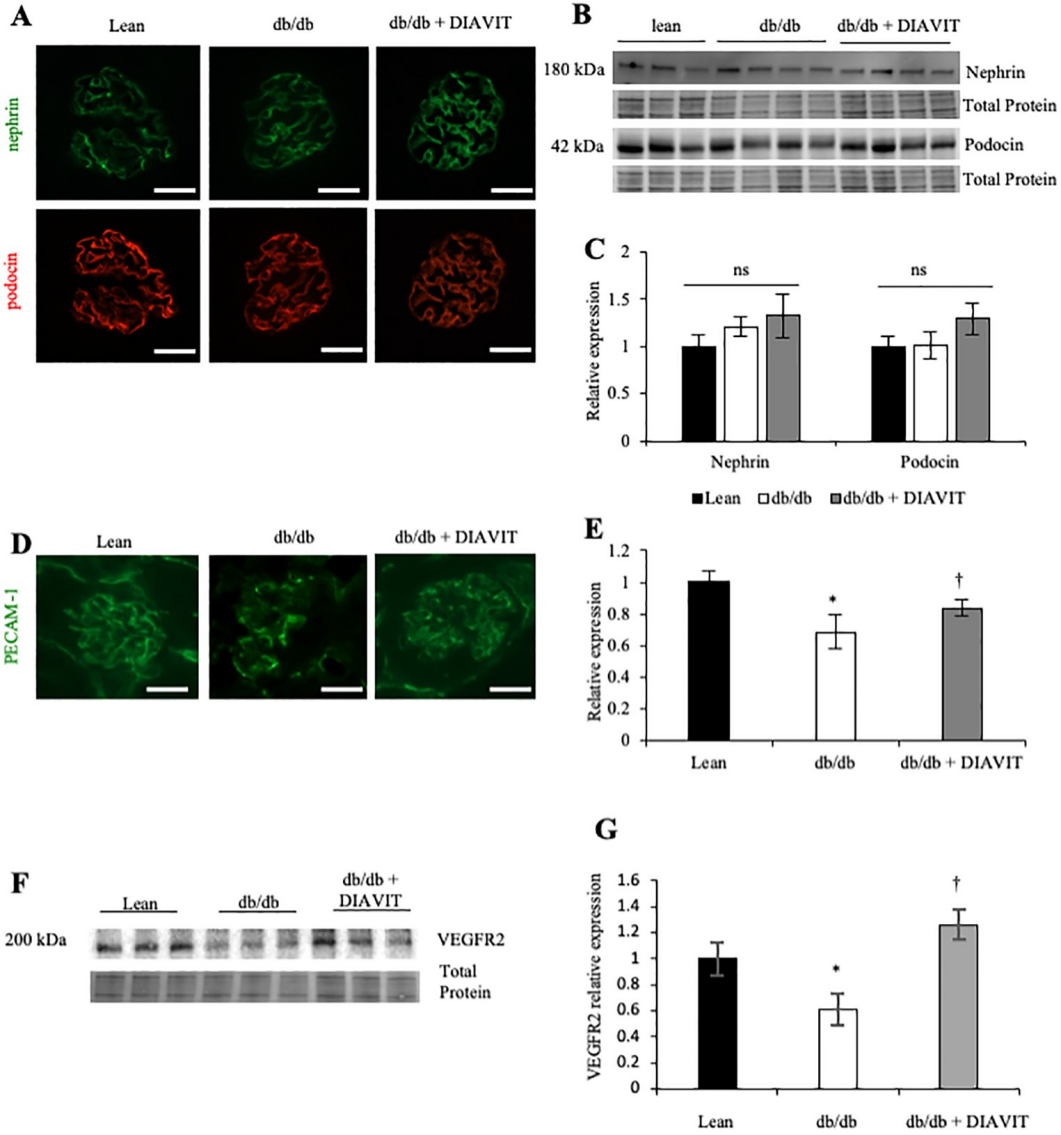
Diabetic db/db mice develop an endothelial insult, which is prevented by DIAVIT treatment of diabetic db/db mice. **A)** Immunofluorescence for nephrin and podocin showed no change in the expression of these podocyte markers between all groups (n = 4 mice; scale bar 40 μm). This was confirmed by Western blotting of protein extracted from the kidney cortex **(B)**; analysis is summarised in **(C)** (ns; p>0.05; n = 3–4 mice; One-way ANOVA with Bonferroni post-hoc test for comparison between pairs). **D)** Immunofluorescence for the endothelial marker PECAM-1 showed a reduction in PECAM-1 expression in db/db glomeruli, which was prevented when db/db mice were treated with DIAVIT; analysis shown in **(E)** (*p<0.05 lean vs db/db; †p<0.05 db/db vs db/db + DIAVIT; n = 3–4 mice; 14–20 glomeruli per mouse; One-way ANOVA with Bonferroni post-hoc test for comparison between pairs; scale bar 40 μm). **F)** Western blotting for the endothelial marker VEGF receptor 2 (VEGFR2) showed a decrease in the db/db kidney cortex, further indicating endothelial loss, which was prevented, and even increased relative to controls, in DIAVIT-treated db/db mice. Analysis is shown in **(G)** (*p<0.05 lean vs db/db; †p<0.05 lean and db/db vs db/db + DIAVIT; n = 3–4 mice; One-way ANOVA with Bonferroni post-hoc test for comparison between pairs).

In order to assess how DIAVIT is protective in type II DN, we determined the expression of podocyte and endothelial-specific proteins as markers of cell function/loss. Immunofluorescence for the podocyte markers nephrin and podocin indicated no changes in the expression of these two proteins in any of the groups (Fig 3A); therefore, suggesting no podocyte loss. This was further confirmed by Western blotting of protein from the kidney cortex (Fig 3B and 3C).

Immunofluorescence for the endothelial marker PECAM-1 showed reduced expression in db/db glomeruli relative to lean controls (Fig 3D and 3E; *p<0.05). This was significantly prevented in db/db + DIAVIT glomeruli (†p<0.05). Further evidence for endothelial loss in db/db glomeruli was indicated by reduced protein expression of VEGFR2 in the cortex of db/db mice, as assessed by Western blotting, relative to lean controls (Fig 3F and 3G;*p<0.05). The reduction in VEGFR2 expression was prevented, and even significantly increased relative to controls in the db/db + DIAVIT kidney cortex (†p<0.05). Therefore, DIAVIT prevented the type II diabetes-induced reduction in renal endothelial markers.

### DIAVIT protects against ultra-structural changes

**Fig 4:**
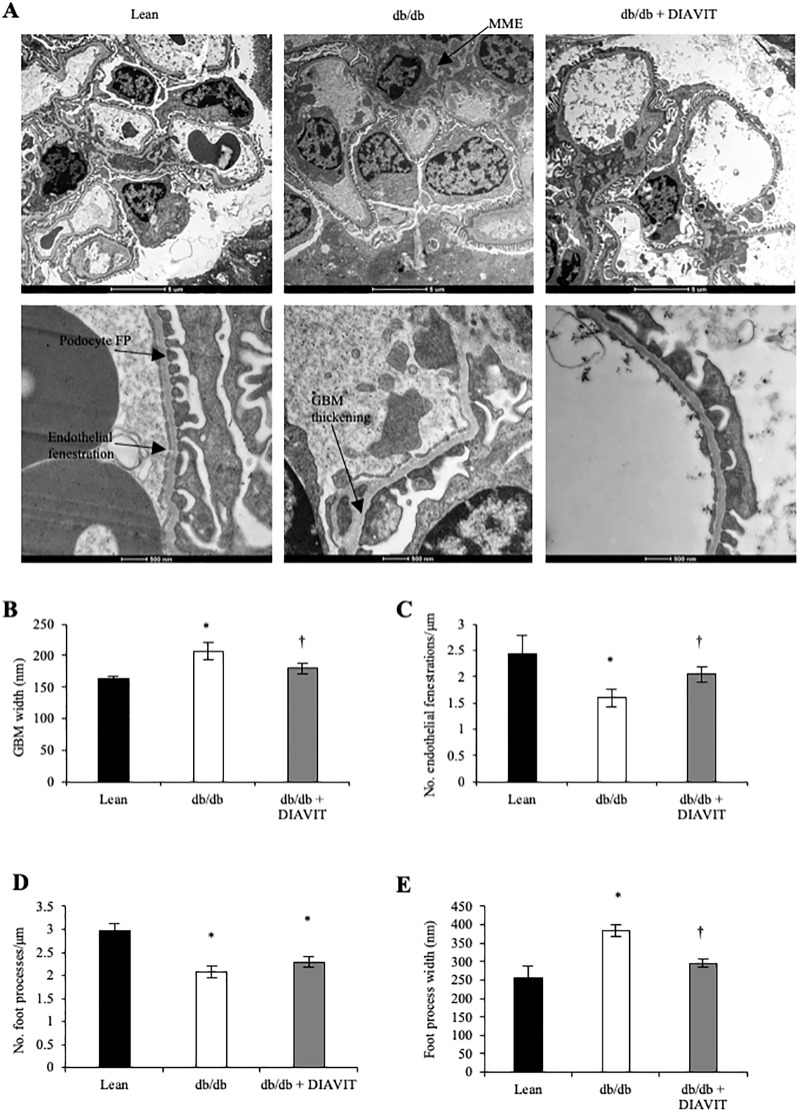
DIAVIT protects against diabetes-induced glomerular ultra-structural changes. **A)** Representative glomerular electron micrographs from lean, db/db, and db/db + DIAVIT mice. Diabetic db/db glomeruli shows evidence of MME, which is not apparent in lean or db/db + DIAVIT glomeruli. Diabetic db/db glomeruli developed an increased GBM width (**B**), decreased number of endothelial fenestrations (**C**) and podocyte foot processes (**D**) per μm length, and an increased average podocyte foot process width (**E**) compared to lean controls (*p<0.05 vs lean). DIAVIT prevented the changes to the GBM width (**B**), number of endothelial fenestrations per μm length (**c**), and average podocyte foot process width (**E**) in db/db + DIAVIT glomeruli (†p<0.05 vs db/db). DIAVIT had no effect on the podocyte foot processes per μm length (**D**) (*p<0.05 vs lean) (n = 3 mice; One-way ANOVA with Bonferroni post-hoc test for comparison between pairs).

We investigated the glomerular ultra-structural phenotype using EM. In diabetic db/db mice, there was evidence of MME, which was not apparent in db/db + DIAVIT glomeruli (Fig 4A). In addition, db/db mice developed an increased GBM width, loss of endothelial fenestrations, reduced number of podocyte foot processes, and an increased podocyte foot process width (Fig 4B and 4E; *p<0.05). However, in db/db + DIAVIT glomeruli, the diabetes-induced changes in the GBM, number of endothelial fenestrations, and podocyte foot process width were prevented (†p<0.05). Therefore, DIAVIT protects against diabetes-induced glomerular ultra-structural changes.

### DIAVIT alters VEGF-A splicing

**Fig 5:**
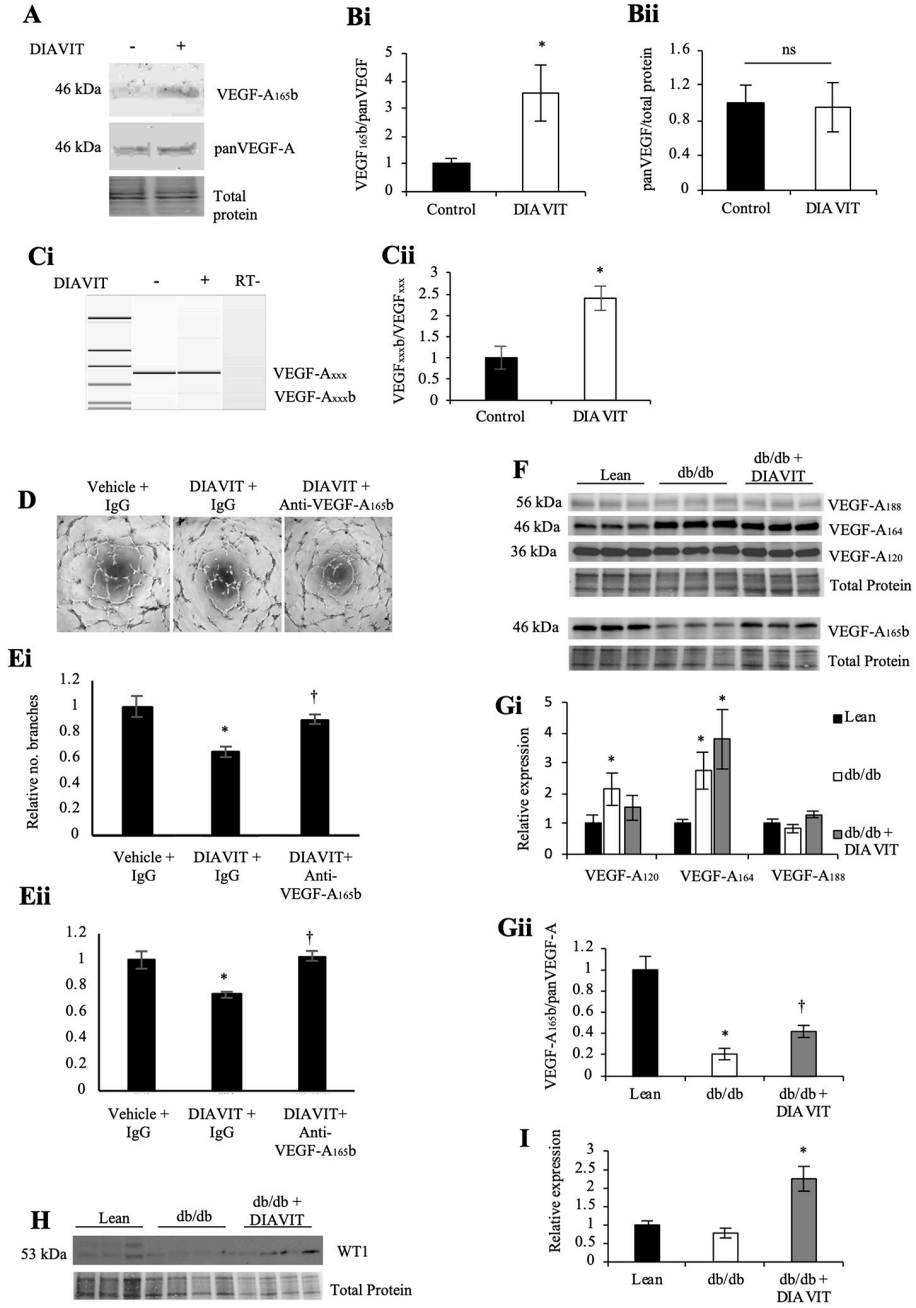
DIAVIT alters VEGF-A splicing to increase VEGF-A_165_b. **A)** DIAVIT treatment (1 mg/ml) of podocytes for 48 hrs resulted in an increased protein expression of VEGF-A_165_b relative to total VEGF-A_165_ (quantified in **B**; *p<0.05; n = 3 biological repeats; T-test; **A**—the same blot was first probed with VEGF-A_165_b before stripping and reprobing with panVEGF-A). **C)** This switch in splicing to increase the VEGF-A_xxx_b/VEGF-A_xxx_ ratio was also observed at the mRNA level (*p<0.05; n = 4 biological repeats; T-test). **D**) DIAVIT inhibited angiogenesis in HUVECs when plated on to Matrigel (*p<0.05 vs control), which was partially prevented when an antibody specific for VEGF-A_165_b was added to the treatment (†p<0.05 vs DIAVIT). Measurments were taken in the form of the relative number of branch points (**Ei**) and the relative tubule length (**Eii**) (n = 4; One-way ANOVA with Bonferroni post-hoc test for comparison between pairs). **F)** Diabetic db/db mice showed switches in the spicing of VEGF-A in the kidney cortex; VEGF-A_120_ and VEGF-A_164_ were increased relative to lean controls (**Gi**; *p<0.05 and *p<0.01, VEGF-A_120_ and VEGF-A_164_, respectively; all VEGF-A isoforms were detected on the same blot), whereas VEGF-A_165_b was down-regulated (**Gii**; *p<0.05). Treatment of the diabetic mice with DIAVIT resulted in no significant increases in VEGF-A_120_ (**Gi**); although no effect was observed on total VEGF-A_164_ expression compared to un-treated db/db mice, DIAVIT did cause a shift in splicing to up-regulate VEGF-A_165_b relative to VEGF-A_164_ (**Gii**; †p<0.05 vs diabetic; n = 3–6 mice; One-way ANOVA with Bonferroni post-hoc test for comparison between pairs). **H)** Diabetes did not alter the expression of the podocyte marker and transcription factor WT1. However, DIAVIT did result in an increase in WT1 expression in the kidney cortex of db/db mice **(I)** (*p<0.05; n = 4 mice; One-way ANOVA with Bonferroni post-hoc test for comparison between pairs).

We assessed the protein expression of VEGF-A, which is also highly implicated in DN, and the alternatively spliced anti-angiogenic isoform VEGF-A_165_b. Treatment of conditionally immortalized podocytes with DIAVIT (1 mg/ml) for 48 hrs resulted in a splicing switch to increase the protein expression of VEGF-A_165_b relative to total VEGF-A_165_ (Fig 5A, quantified in Fig 5Bi; *p<0.05), with no effect on pan-VEGF-A expression (Fig 5Bii). We also saw a significant increase in the VEGF-A_xxx_b/VEGF-A_xxx_ ratio at the mRNA level via RT-PCR, quantified using a bioanalyzer (Fig 5C). Therefore, we assessed the effects of DIAVIT on angiogenesis using a Matrigel angiogenesis assay. HUVECs were plated onto Matrigel and treated with conditioned media from podocytes pre-treated with DIAVIT for 48 hrs. Four hours later, DIAVIT-treated HUVECs had a reduction in the relative number of branch points and the relative tubule length (Fig 5D and 5E; *p<0.05), compared to untreated controls. This anti-angiogenic effect was significantly reversed when the podocytes and HUVECs were treated with DIAVIT and an antibody specific for the VEGF-A_165_b isoform (†p<0.05).

When analyzing the splicing pattern of VEGF-A in the kidney cortex of diabetic db/db mice, there was a significant increase in the relative expression of VEGF-A_120_, which was not observed in DIAVIT-treated db/db mice (Fig 5F and 5G; *p<0.05). An increase in the expression of VEGF-A_164_ (which corresponds to human VEGF-A_165_) was also observed in both diabetic groups relative to lean controls (*p<0.01). No changes in the expression of VEGF-A_188_ was observed in either group. When looking specifically at the splicing VEGF-A_164_, the anti-angiogenic VEGF-A_165_b (alternatively spliced isoform of VEGF-A_164_) was down-regulated in the db/db kidney cortex relative to lean controls (*p<0.05). This was partially prevented when the db/db mice were treated with DIAVIT (†p<0.05). Therefore, DIAVIT switches VEGF-A_165/164_ splicing to increase the anti-angiogenic VEGF-A_165_b isoform.

The expression of the transcription factor WT1, which promotes VEGF-A_165_b splicing [25], remains unchanged in the kidney cortex in diabetic db/db mice relative to controls; however, DIAVIT treatment induced a significant increase in WT1 expression (Fig 5H and 5I; relative expression: db/db + DIAVIT, *p<0.05).

### DIAVIT prevents the activation of pro-angiogenic and pro-fibrotic factors

**Fig 6:**
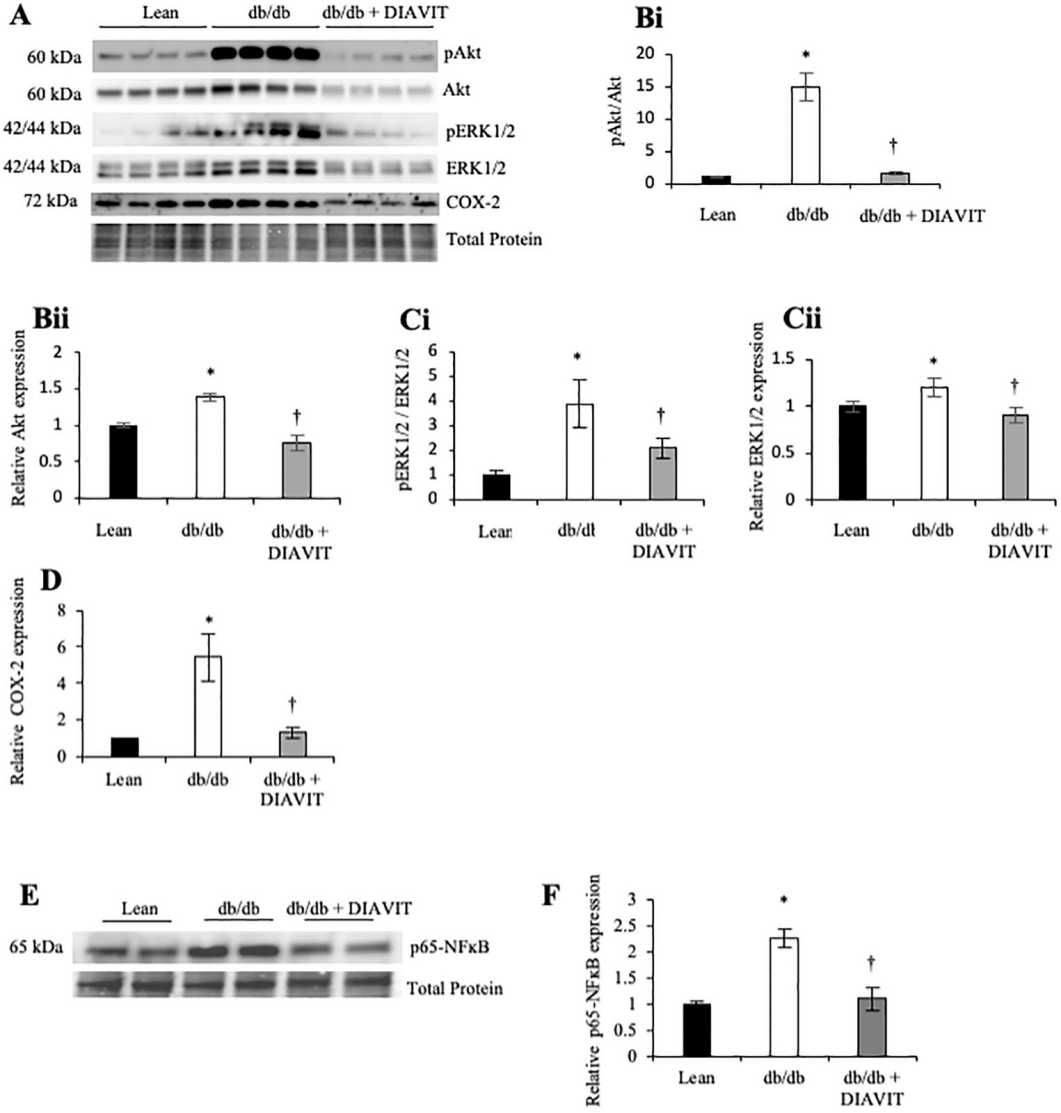
Diabetic mice develop an increase in the activation of pro-angiogenic and pro-fibrotic factors in the kidney, which is prevented with DIAVIT treatment. **A)** Western blotting of protein extracted from the kidney cortex shows an increased phosphorylation and expression of Akt^Ser473^ (**A, B**), an increase in the phosphorylation and expression of ERK1/2 (**a, c**), and an increase in the expression of COX-2 in diabetic db/db mice (**A, D**; phosphorylation *p<0.01; expression *p<0.05 vs lean). DIAVIT prevented the increased activation and expression of these factors in the diabetic mice (**A-D**; †p<0.05 vs diabetic; n = 3–8 mice; all proteins were detected on the same blot). In addition, diabetic db/db mice had an increased expression of p65-NFĸB in the kidney cortex, which was singifcantly rescued by DIAVIT treatment (**E, F**; p<0.05; n = 3–4 mice; One-way ANOVA with Bonferroni post-hoc test for comparison between pairs).

To further assess the effects of DIAVIT on the diabetic kidney, we looked at the activation of pro-angiogenic/permeability factors known to be involved in the progression of DN. We found the phosphorylation of Akt^Ser473^ and ERK1/2, normalized to total Akt and ERK1/2, respectively, to be increased in the kidney cortex of diabetic db/db mice (*p<0.01); the relative expression of Akt, ERK1/2 and COX-2 was also increased (Fig 6A, 6B, 6C and 6D; *p<0.05). All of the above was prevented in diabetic db/db + DIAVIT mice (†p<0.05) (Fig 6A, 6B, 6C and 6D).

We also assessed the effects of DIAVIT on the protein expression and activation of p65-NFĸB, which is involved in pro-fibrotic gene transcription [13]. In the db/db kidney cortex, after 14 weeks of diabetes, there was a significant increase in the protein expression of p65-NFĸB, relative to lean controls (Fig 6E and 6F; *p<0.05). This increase in p65-NFĸB was prevented by DIAVIT treatment of the diabetic db/db mice (†p<0.05).

### Delphinidin alters the expression and splicing ratio of VEGF-A in podocytes

**Fig 7:**
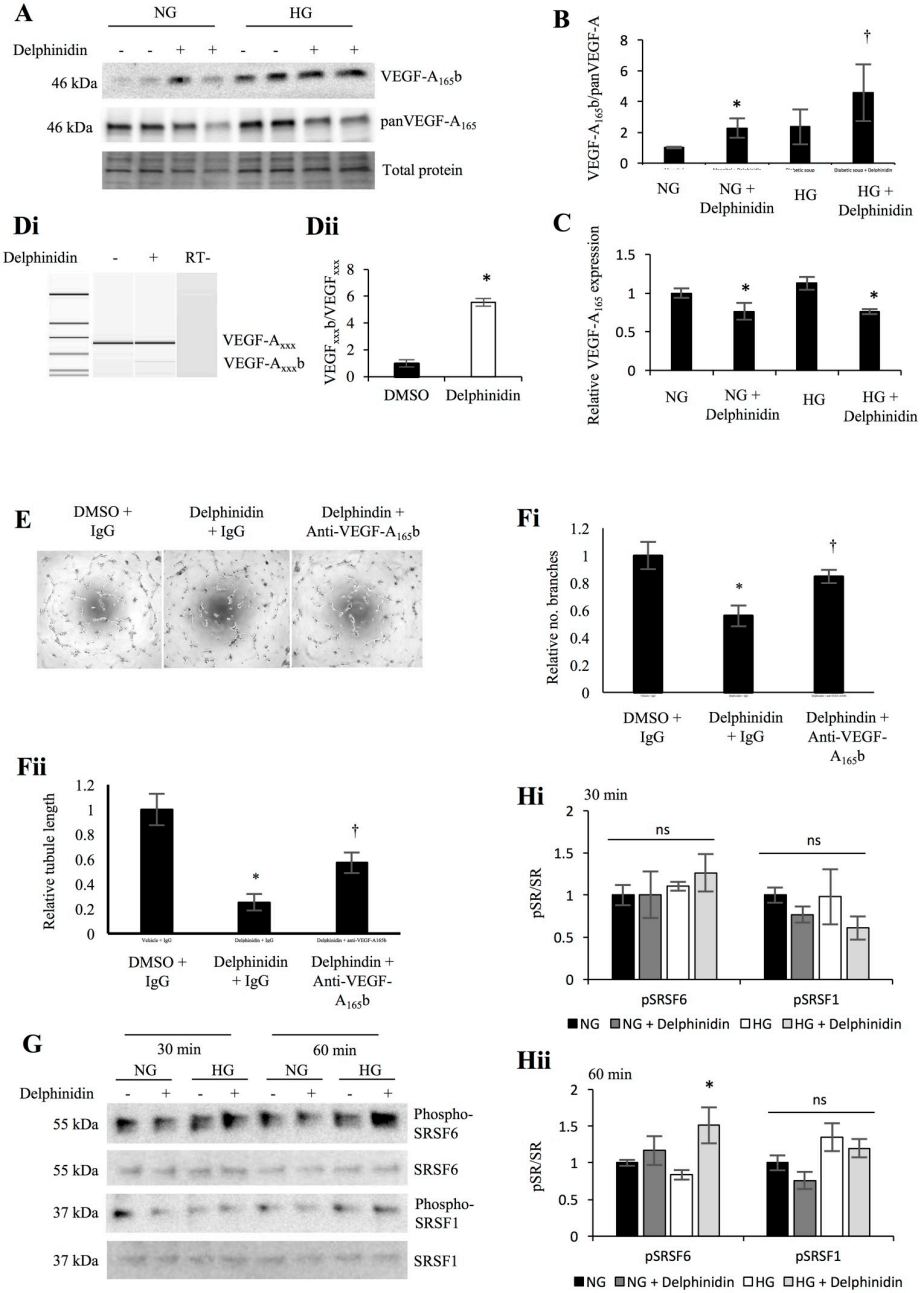
Delphinidin alters VEGF-A splicing to increase VEGF-A_165_b and decrease total VEGF-A expression. **A)** Treatment of podocytes with delphinidin chloride (10 μg/ml) under normal glucose (NG; 5 mM glucose + 25 mM mannitol) and high glucose (HG; 30 mM glucose, 1 ng/ml TNFα, 1 ng/ml IL-6, and 100 nM insulin) for 48 hrs increased the protein expression of VEGF-A_165_b relative to total VEGF-A_165_ (quantified in **B**; *p<0.05 vs NG, †p<0.05 vs HG; n = 3 biological repeats; One-way ANOVA with Bonferroni post-hoc test for comparison between pairs; **A**—the same blot was first probed with VEGF-A_165_b before stripping and reprobing with panVEGF-A). **C)** Under both NG and HG condition, delphinidin significantly decreased the protein expression of total VEGF-A_165_ (*p<0.05; n = 3 biological repeats; One-way ANOVA with Bonferroni post-hoc test for comparison between pairs). **D)** Analysis at the mRNA level shows an increase in the VEGF-A_xxx_b/VEGF-A_xxx_ ratio after treatement with delphinidn (*p<0.05; n = 4 biological repeats; T-test). **E)** Delphinidin inhibited angiogenesis in HUVECs when plated on to Matrigel (*p<0.05 vs control). Addition of an antibody specific for VEGF-A_165_b did not alter the effect of delphinidin. Measurments were taken in the form of the relative number of branch points (**Fi**) and the relative tubule length (**Fii**) (n = 4; One-way ANOVA with Bonferroni post-hoc test for comparison between pairs). **G)** Cells pre-treated with DMSO/delphinidin were stimulated with NG/HG for 30 or 60 mins. After 30 min, there was no significant effect of delphinidin on the phosphorylation of SRSF6 or SRSF1 under either condition **(H)**. After 60 min, delphinidin significantly increased the phosphorylation of SRSF6 after stimulation with HG (*p<0.05 vs NG and HG controls; n = 3 biological repeats; One-way ANOVA with Bonferroni post-hoc test for comparison between pairs). There was no significant effect on phospho-SRSF1 at 60 min.

We further assessed the mechanistic effects of the most abundant anothocyanin in DIAVIT, delphinidin, on VEGF-A splicing and expression. Treatment of conditionally immortalized podocytes with delphinidin (10 μg/ml) for 48 hrs resulted in a splicing switch to increase VEGF-A_165_b relative to total VEGF-A_165_ in both normal glucose (NG) and high glucose (HG) conditions (protein: Fig 7A, quantified in Fig 7B; p<0.05; mRNA: Fig 7D). In addition, delphinidin significantly decreased the expression of total VEGF-A_165_ (Fig 7C; p<0.05). Therefore, we assessed the effects of delphinidin on angiogenesis using a Matrigel angiogenesis assay. HUVECs were plated onto Matrigel and treated with conditioned media from podocytes pre-treated with delphinidin for 48 hrs. Four hours later, delphinidin-treated HUVECs had a reduction in the relative number of branch points and the relative tubule length (Fig 7E and 7F; *p<0.05), compared to DMSO treated controls. This anti-angiogenic effect was not significantly reversed when the podocytes and HUVECs were treated with delphinidin and an antibody specific for the VEGF-A_165_b isoform.

To determine the mechanism of the effect of delphinidin on VEGF-A splicing, we analysed the phosphorylation of SRSF6 (resulting in VEGF-A_165_b expression) and SRSF1 (resulting in VEGF-A_165_ expression) in NG and HG conditions. We found that after 60 min, delphinidin significantly increased the phosphorylation of SRSF6 in HG conditions (Fig 7G, quantified in Fig 7H; p<0.05). Delphinidin had no effect on the phosphorylation of SRSF1 in either condition.

## Discussion

This study provides evidence that DIAVIT, a natural drug containing blueberry and sea buckthorn, is protective in a type II mouse model of DN. In this study, DIAVIT prevented the increased permeability of the GFB, fibrosis in the kidney cortex, and an endothelial insult in type II diabetic mice. Mechanistically, DIAVIT is likely to be affecting multiple pathways as it is comprised of hundreds of compounds. However, with regards to microcirculation/permeability and fibrosis, DIAVIT switched the splicing of VEGF-A to increase the expression of the anti-angiogenic VEGF-A_165_b, and reduced the activation of pro-inflammatory and pro-fibrotic markers. Furthermore, the most abundant anthocyanin in DIAVIT, delphinidin, was found to modulate VEGF-A expression and splicing through the activation of SRSF6. This resulted in an anti-angiogenic effect through up-regulation of the anti-angiogenic VEGF-A_165_b, but a reduction in the expression of total VEGF-A.

With increasing evidence to suggest that DN is driven by microvessel damage and increased permeability, fibrosis, and inflammation, recent developments have focused on therapeutic factors that prevent the up-regulation of these pathways [7,17,18]. VEGF-A is widely accepted to be involved in the vascular complications related to diabetes; however, the alternative splice isoform VEGF-A_165_b has recently proven to be therapeutically beneficial in both type I and type II models of DN [7]. In addition, switches in VEGF-A splicing to up-regulate the VEGF-A_xxx_b (_xxx_ denotes the number of amino acids) isoforms have also been shown to have anti-angiogenic, and therefore therapeutic, effects in models of retinopathy and cancer [29,30]. To our knowledge, this is the first report to suggest an anthocyanin present in a natural drug can switch VEGF-A splicing to promote the expression of the anti-angiogenic VEGF-A_165_b both in the mouse kidney cortex and in conditionally immortalized podocytes. Over-expression of just the VEGF-A_165_b isoform in the podocytes of mice has no detrimental effects on kidney function [31]. Furthermore, over-expression of podocyte-specific VEGF-A_165_b when all other isoforms of VEGF-A are depleted results in a rescue of the glomerular injury phenotype [12]. We show that in db/db mice the splicing and expression of VEGF-A are altered to increase the pro-angiogenic VEGF-A_164_ and decrease the anti-angiogenic VEGF-A_165_b, which is consistent with what is reported in human diabetic nephropathy [7]. However, DIAVIT partially prevents this switch in splicing, resulting in an increase in the VEGF-A_165_b/VEGF-A_164_ ratio in the diabetic mice (Fig 5E). We hypothesize that this switch in splicing contributed to the therapeutic phenotype induced by DIAVIT, including preventing increases in albuminuria and glomerular water permeability (Fig 1), glomerular fibrosis (Fig 2), and the ultra-structural changes to the mesangial cells, endothelial cells, GBM, and podocyte foot processes (Fig 4), which have previously been shown to be rescued by VEGF-A_165_b treatment in models of DN and kidney disease [7,12].

VEGF-A_165_b has been reported to increase the glomerular endothelial cell VEGFR2 expression [7,12]. We show a similar result; the kidney cortex expression of VEGFR2 is decreased in diabetic mice, which is rescued to expression levels higher than the lean controls in db/db + DIAVIT mice (Fig 3F). This is likely to be in part due to the increased expression of VEGF-A_165_b in the DIAVIT-treated mice. Although VEGF-A_165_b increases the expression of VEGFR2, it inhibits signalling by preventing receptor phosphorylation, as previously shown in HUVECs [30], and ciGEnCs [12]. As a result, the activation of pro-angiogenic and pro-permeability proteins downstream of VEGFR2, such as Akt and ERK1/2, are inhibited [32]. We see a similar result in this study; diabetic mice treated with DIAVIT have reduced phosphorylation and expression of Akt and ERK1/2 compared to diabetic controls (Fig 6A).

Furthermore, activation of Akt has been previously reported to result in the auto-phosphorylation of serine/threonine-protein kinase 1 (SRPK1), an SR protein kinase responsible for phosphorylating SRSF1, which promotes proximal splice site selection in exon 8 of VEGF-A and thus increases the expression of the pro-angiogenic VEGF-A_xxx_ isoforms [33]. WT1 is an inhibitor of SRPK1 phosphorylation; Denys Drash Syndrome patients have a mutation in WT1, resulting in no inhibition of SRPK1 and an increased VEGF-A_165_/VEGF-A_165_b ratio [29]. However, the WT1-SRPK1 signaling pathway is not the only pathway known to regulate VEGF-A splicing; SRSF6, which promotes distal splice site selection in exon 8 of VEGF-A (increasing VEGF-A_xxx_b expression) has recently been shown to be down-regulated in diabetes [34]. This may be explain why we do not see a change in WT1 expression in db/db mice relative to lean controls, even though the expression of VEGF-A_165_b is decreased in db/db mice. In our study, we see an increase in the phosphorylation of Akt within the kidney cortex of diabetic db/db mice, which is not observed in db/db + DIAVIT mice. Furthermore, we also see an increase in WT1 expression with DIAVIT treatment.

Although there are no reports on delphinidin, or any other anothocyanins, having an effect on gene splicing, delphinidin is widely reported to reduce total VEGF-A expression through the PKC/PI3K/Akt/mTOR signaling pathways, without having any effect on the p38MAPK pathway [24,25]. Previous studies on VEGF-A splicing have shown that upregulation of the PKC pathway results in SRSF1 phosphorylation and proximal splice site selection in exon 8 of VEGF-A, whereas upregulation of the p38MAPK signalling pathways instead induces SRSF6 phosphorylation promoting distal splice site selection [35]. The present study confirms these findings at the level of VEGF-A splicing regulation; delphinidin induced the phosphorylation of SRSF6 but not SRSF1 in podocytes, resulting in an increase in VEGF-A_165_b relative to VEGF-A_165_. Together, this data suggests that DIAVIT, including delphinidin present in DIAVIT, is increasing the VEGF-A_165_b/VEGF-A_165_ ratio through the inhibition of Akt, up-regulation of WT1, and increased phosphorylation of SRSF6.

In addition, we show that delphinidin results in a down-regulation of pro-angiogenic VEGF-A expression, resulting in the inhibition of angiogenesis (Fig 7D), as reported in multiple studies assessing the effects of delphinidin on VEGF-A expression [24,25]. Although the anti-angiogenic effect of DIAVIT was reversed when anti-VEGF-A_165_b was added to the Matrigel assay, leading us to conclude that the total anti-angiogenic effect of DIAVIT was partially the result of alternative splicing, there was no significant reversal of the inhibition of angiogenesis by delphinidin with anti-VEGF-A_165_b. We clearly show that delphinidin promotes VEGF-A_165_b expression relative to VEGF-A_165_; however, delphinidin also has an inhibitory effect on VEGF-A total expression, which is not observed in response to the DIAVIT extract. Therefore, we postulate that although delphinidin induces a splicing switch to promote the anti-angiogenic VEGF-A isoform expression, it is the inhibitory effect on VEGF-A expression that is the main factor contributing to its anti-angiogenic effect. Furthermore, delphinidin has been previous reported to be cytoprotective to endothelial function [36]. This may explain why we see a reno-protective effect of DIAVIT without any effect on blood glucose levels.

**Fig 8:**
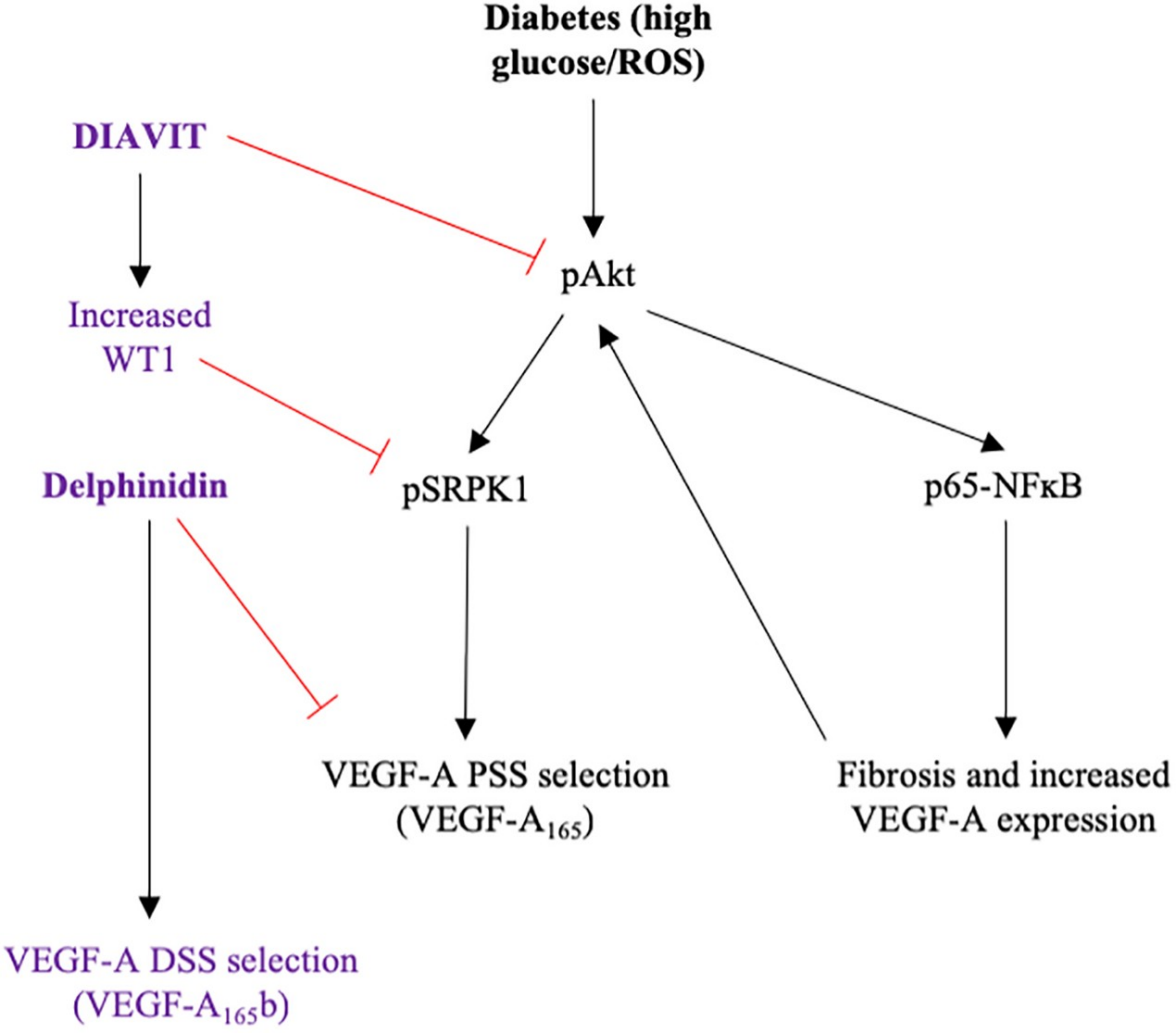
Flow diagram describing the potential mechanism of DIAVIT and Delphinidin in type II DN. High glucose and an increase in oxidative stress (ROS; reactive oxygen species) in the kidney results in increased phosphorylation of Akt. In turn this leads to activation of p65-NFĸB, resulting in the increased expression of pro-angiogenic and pro-fibrotic factors, which feed back to further increase the activation of Akt. Akt activation also results in the phosphorylation of serine protein kinase 1 (SRPK1), leading to proximal splice site (PSS) selection in exon 8 of the VEGF-A gene (pro-angiogenic VEGF-A_165_). DIAVIT acts to inhibit the phosphorylation of Akt and increase the expression of the SRPK1 inhibitor WT1. Furthermore, delphinidin acts to switch VEGF-A splicing, promoting distal splice site selection through the activation of SRSF6. This results in a reduction in fibrosis and angiogenesis in the diabetic kidney through p65-NFĸB and VEGF-A splicing.

In DN, glucotoxicity results in the increased generation of free radicals and oxidative stress [15]. Activation of NFĸB is widely reported to be evoked by increased oxidative stress [16]. Previous studies in rodent models of DN have indicated that a reduction in oxidative stress using anti-oxidants, such as those found in red berry extracts, resulted in decreased NFĸB activity, thus improving kidney function [17,18]. We show that DIAVIT prevented the increases in NFĸB expression within the kidney cortex of db/db mice, resulting in an inhibition of glomerular fibrosis and endothelial injury (Fig 6G). Furthermore, anothocyanins have been reported to protect against fibrosis, angiogenesis, and inflammation in the kidneys of diabetic animal models [37,38]. We summarize the potential mechanism of action of DIAVIT and delphinidin in DN using a flow diagram in Fig 8.

One limitation of this study was that we were unable to fully control the dose of DIAVIT given to the mice as it was added to the drinking water. Diabetic mice, on average, drank more water than the lean controls; as the levels of hyperglycaemia were similar in both db/db and db/db + DIAVIT mice, we estimated that they consumed approximately the same amount water.

## Conclusions

In conclusion, we show that DIAVIT prevents increases in permeability and fibrosis in a mouse model of type II DN. One of the mechanisms by which DIAVIT, namely delphinidin, is acting are through switching VEGF-A splicing in the podocytes of the renal cortex. This study highlights the therapeutic potential of natural drugs and anthocyanins in DN through the manipulation of gene splicing and expression. Further studies are required to further deduce which other compound(s) in DIAVIT are having similar effects.

## Supporting information

S1 Fig Gas chromatography mass spectrometry (GC-MS) of the DIAVIT extract. To determine the chemical composition of the DIAVIT extract, we performed GC-MS, which generated the chromatogram observed in **(A)**. **(B)** Mass spectrometry was carried out on each peak to sort the ions based on their mass-to-charge ratio. The extract was found to be extremely complex, with some chemical examples given.
(PDF)

S2 Fig Absolute urinary albumin creatinine ratio (uACR) values. *p<0.05 lean vs db/db, †p<0.05 db/db vs db/db + DIAVIT; Two-way ANOVA.
(TIFF)
