# Supplementary figures and images for: The natural drug DIAVIT is protective in a type II mouse model of diabetic nephropathy
Source: PLoS One. 2019 Mar 13;14(3):e0212910. doi: 10.1371/journal.pone.0212910 (PMC6415805; doi:10.1371/journal.pone.0212910)

S1 A

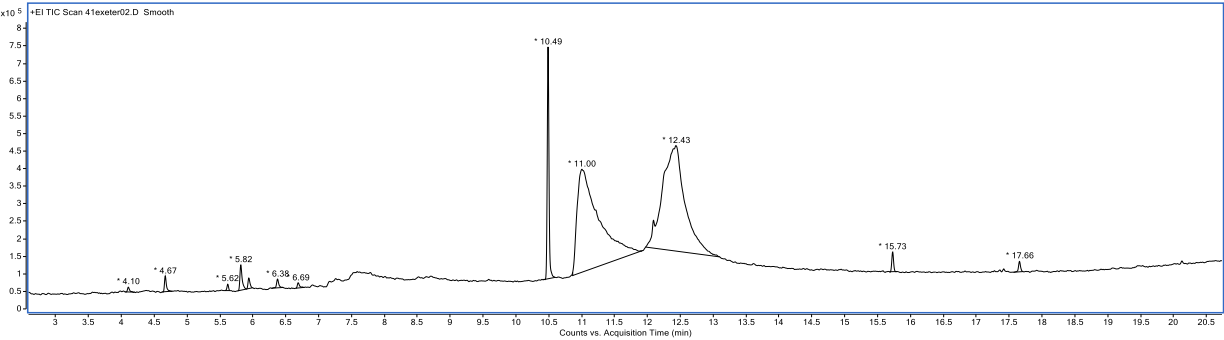

S1 B

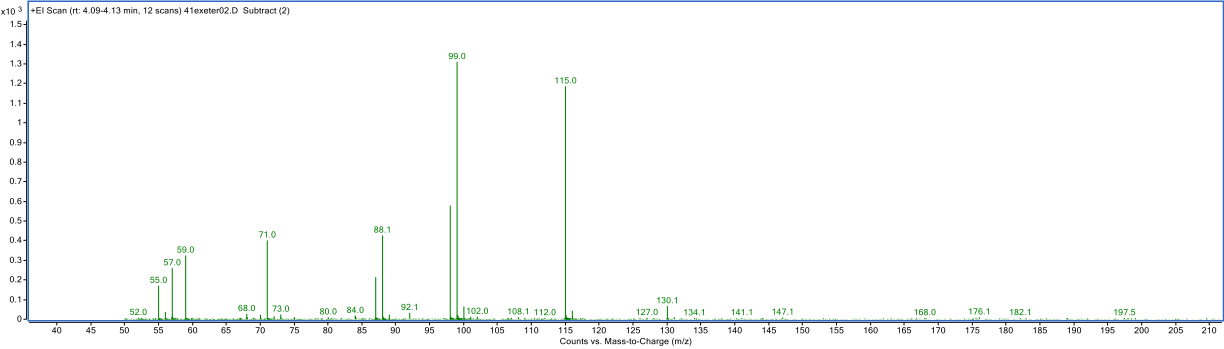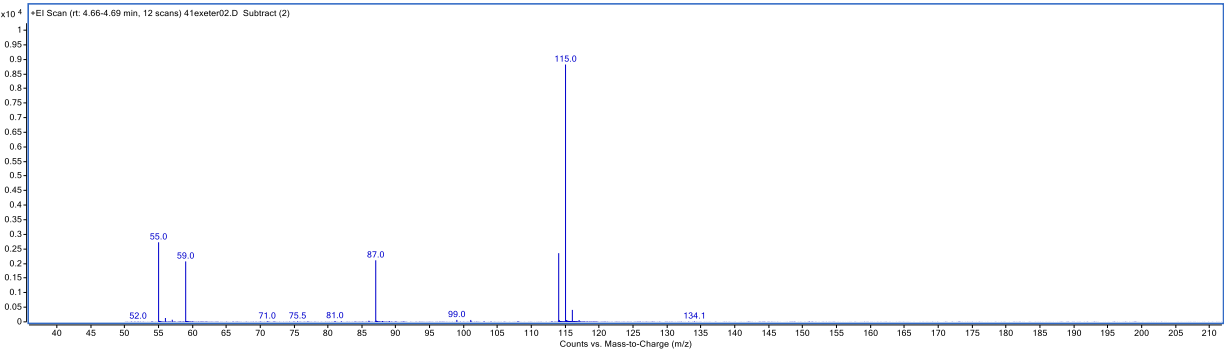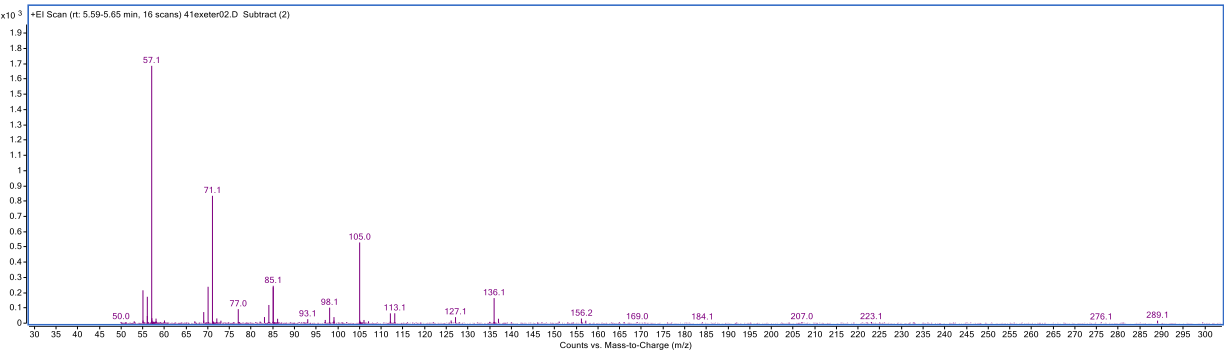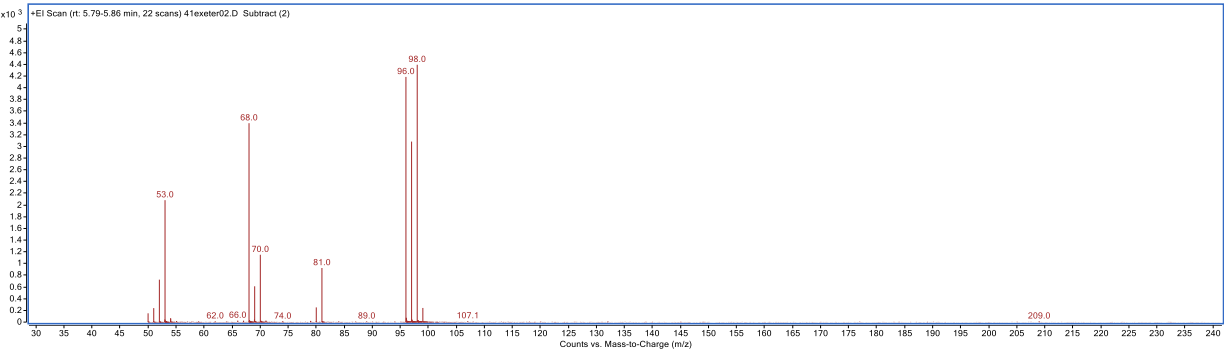

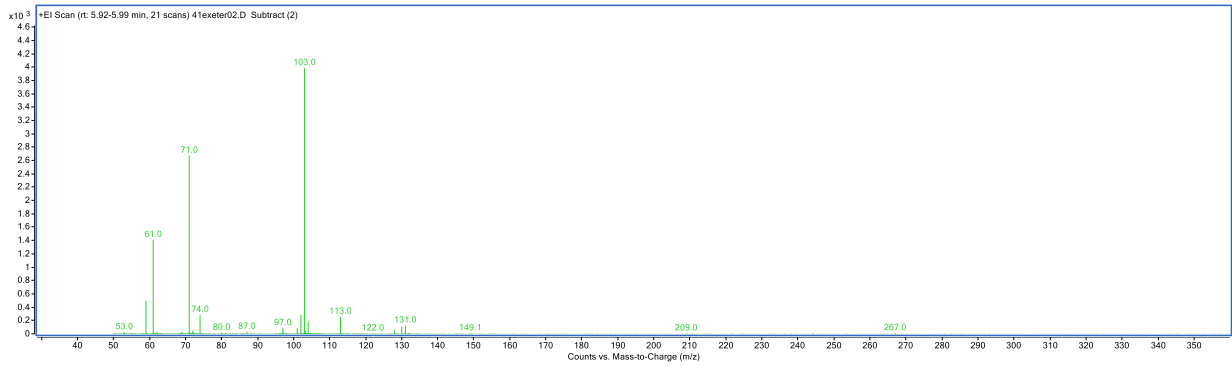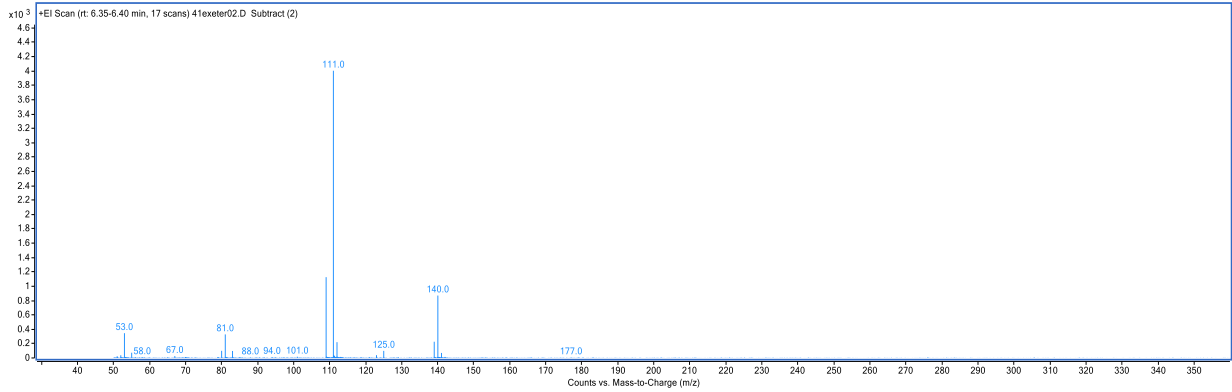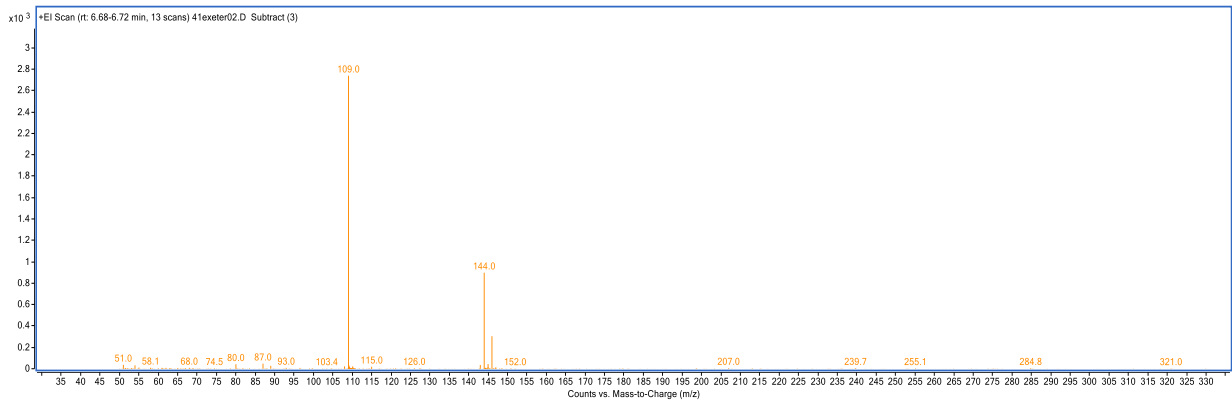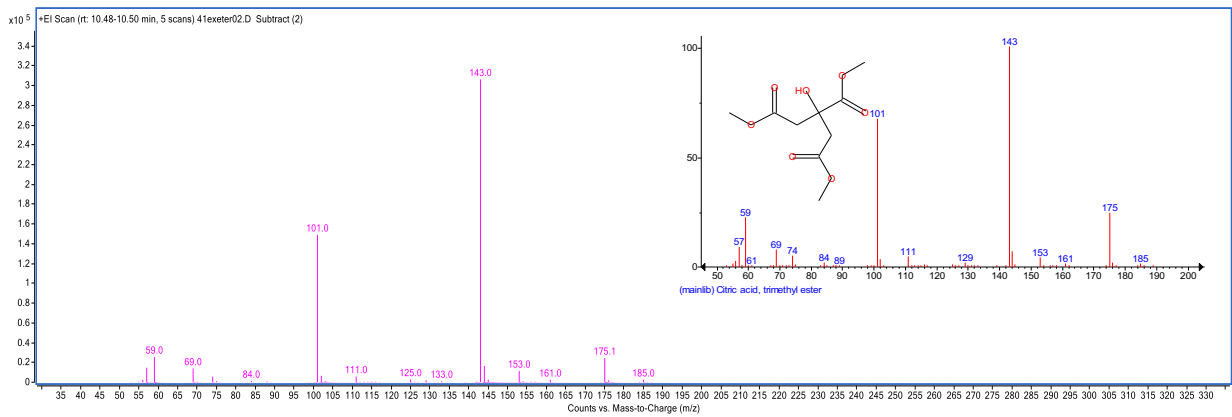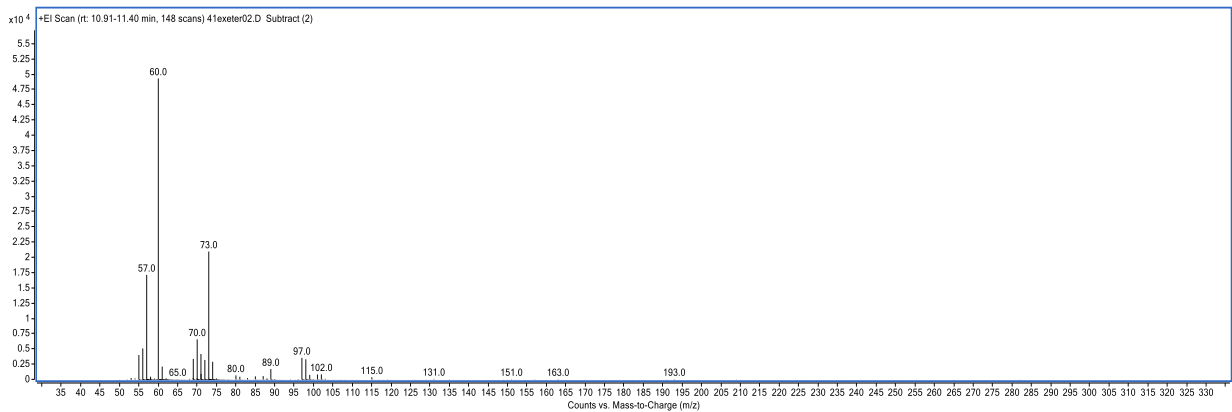

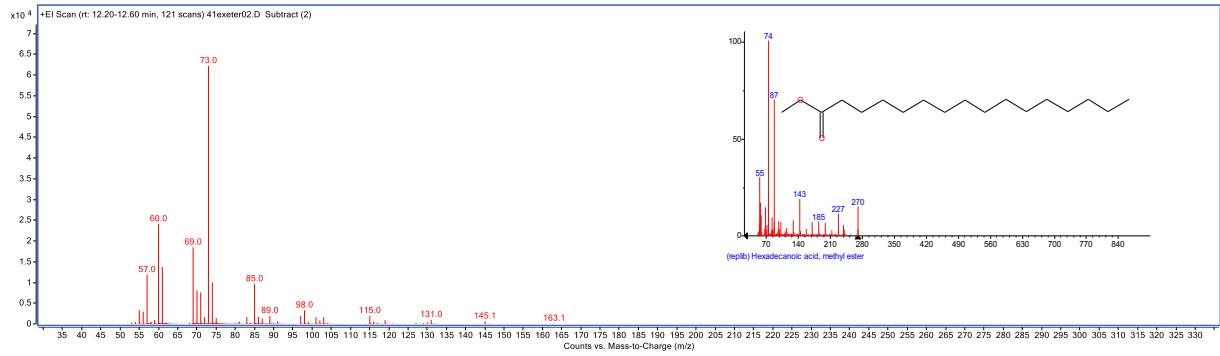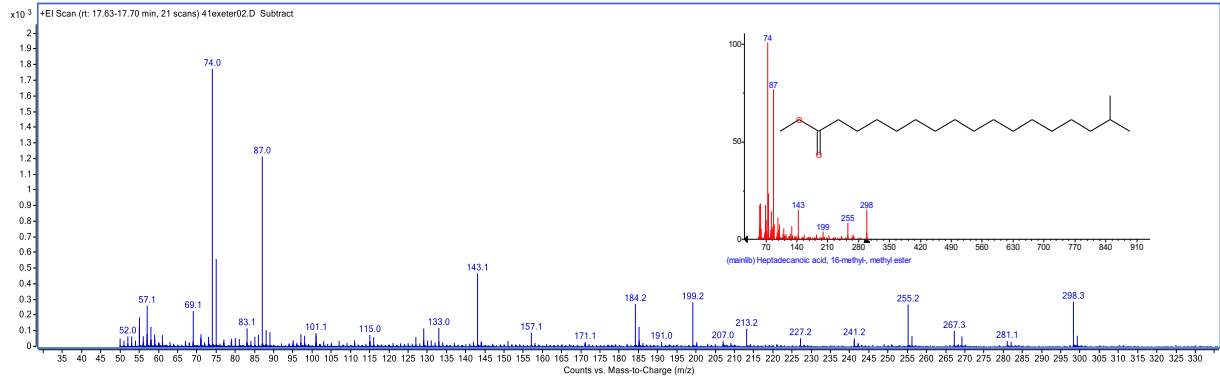

Supplement: S1 Fig — To determine the chemical composition of the DIAVIT extract, we performed GC-MS, which generated the chromatogram observed in (A). (B) Mass spectrometry was carried out on each peak to sort the ions based on their mass-to-charge ratio. The extract was found to be extremely complex, with some chemical examples given. (PDF) [file pone.0212910.s001.pdf]

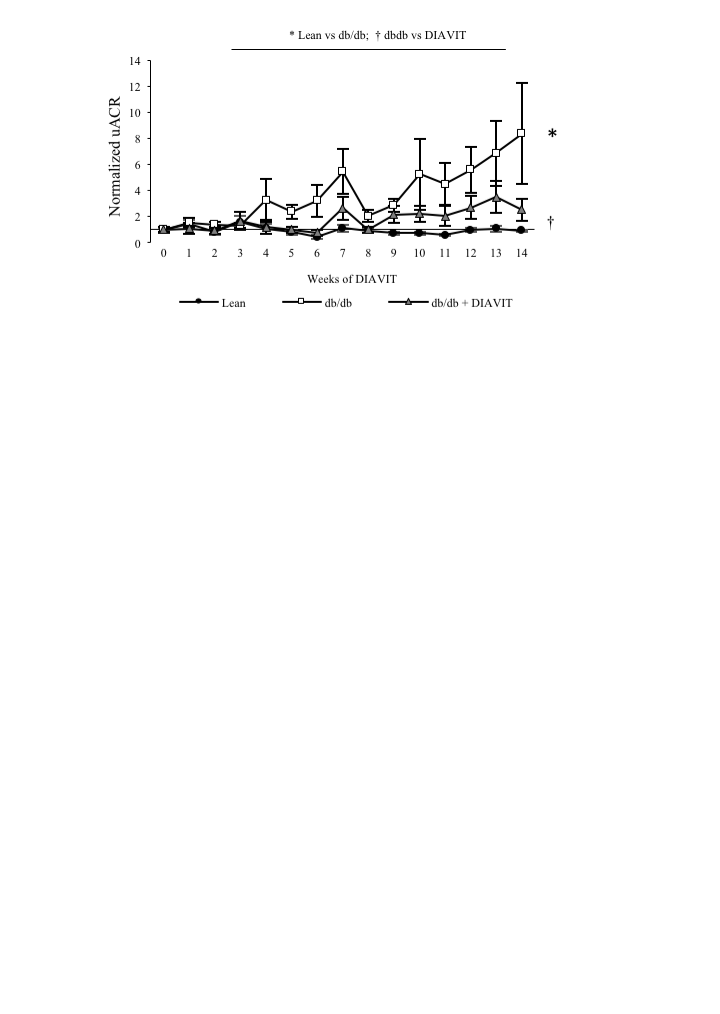

Supplement: S2 Fig — *p<0.05 lean vs db/db, †p<0.05 db/db vs db/db + DIAVIT; Two-way ANOVA. (TIFF) [file pone.0212910.s002.tiff]
